# Recent advances in osteonecrosis of the femoral head: a focus on mesenchymal stem cells and adipocytes

**DOI:** 10.1186/s12967-025-06564-6

**Published:** 2025-05-27

**Authors:** Shilei Zhang, Haojue Wang, Qi Meng, Wayne Yuk-wai Lee, Ziqing Li, Shui Sun

**Affiliations:** 1 Department of Joint Surgery, Shandong Provincial Hospital Affiliated to Shandong First Medical University, Jinan, 250021 Shandong China; 2 Department of Joint Surgery, Cheeloo College of Medicine, Shandong Provincial Hospital, Shandong University, Jinan, 250012 Shandong China; 3 Orthopaedic Research Laboratory, Medical Science and Technology Innovation Center, Shandong First Medical University & Shandong Academy of Medical Sciences, Jinan, 250117 Shandong China; 4 Department of Orthopaedics and Traumatology, The Chinese University of Hong Kong, Hong Kong, China; 5 SH Ho Scoliosis Research Laboratory, Joint Scoliosis Research Centre of the Chinese University of Hong Kong and Nanjing University, The Chinese University of Hong Kong, Hong Kong, China; 6 Li Ka Shing Institute of Health Sciences, The Chinese University of Hong Kong, Hong Kong, China

**Keywords:** Osteonecrosis of the femoral head, Mesenchymal stem cell, Adipocyte, Signaling pathways, Adipokine

## Abstract

Osteonecrosis of the femoral head (ONFH) is a debilitating orthopedic disease characterized by femoral head collapse and destruction of bone and articular cartilage, resulting in severe joint pain and loss of hip mobility. Bone marrow mesenchymal stem cells (BMSCs) exhibit multilineage differentiation potential, including osteoblasts, adipocytes, fibroblasts, chondrocytes, and neurocytes. The imbalance between osteogenesis and adipogenesis in BMSCs plays a critical role in ONFH pathogenesis. Factors such as alcohol consumption and glucocorticoid exposure promote adipogenic differentiation while inhibiting osteogenic differentiation, leading to excessive adipocyte accumulation, reduced bone formation, and vascular impairment. We highlight the molecular mechanisms underlying ONFH with a particular focus on the role of BMSCs and further discuss the involvement of adipocytes. Moreover, we suggest that the use of adipose-derived mesenchymal stem cells (ADMSCs) is a viable approach for stem cell therapy and may have immense potential in ONFH. Several signaling pathways, including the Wnt, TGFβ/BMP, and PI3K/AKT pathways, along with various RNAs and other regulators, govern the osteogenesis and adipogenesis of BMSCs. These signaling pathways target essential transcription factors, such as Runx2 for osteogenesis and PPARγ and C/EBPs for adipogenesis. Adipocytes and their secreted adipokines, including leptin and adiponectin, strongly influence ONFH progression. Emerging therapies involving ADMSCs show potential for promoting bone regeneration and neovascularization. Our review provides a comprehensive overview of the current understanding of ONFH mechanisms by focusing on mesenchymal stem cells and adipocytes and suggests future research directions for therapeutic interventions.

## Introduction

Osteonecrosis of the femoral head (ONFH) is a prevalent yet persistent orthopedic disease characterized by the collapse of the femoral head, destruction of bone and articular cartilage, severe joint pain, and loss of normal hip function [1]. Globally, ONFH affects approximately 30 million people, including approximately 4 million individuals in China [2]. ONFH is generally classified into traumatic and nontraumatic types [3]. The former is associated with femoral neck fractures, hip dislocation and other types of hip trauma [4], whereas the latter is associated with factors such as corticosteroid medication, alcohol consumption and several autoimmune diseases [5]. Regardless of etiology, a compromised blood supply to the femoral head is a major contributor to ONFH [6], and studies suggest that the pathogenesis of ONFH is closely related to severe degradation of bone tissue, increased differentiation of bone mesenchymal stem cells (BMSCs) into adipocytes and/or adipocyte hypertrophy through increased intracellular lipid synthesis [7]. More specifically, the expansion of fatty marrow, together with the subsequent increase in intraosseous pressure, can induce intraosseous venous stasis and hypertension, thereby decreasing arterial perfusion and obstructing the venous system within a semi-intact bony compartment [8, 9]. These pathological changes are postulated to predominantly affect the behaviors of mesenchymal stem cells (MSCs) [10–12].

Stem cells exhibit a self-replicative capacity and multidifferentiation potential due to their undifferentiated or poorly differentiated state [2]. As one of the common stem cell types, BMSCs can differentiate into many lineages, such as osteoblasts, adipocytes, fibroblasts, chondrocytes, and neurocytes [3]. The balance between adipogenic and osteogenic differentiation of BMSCs is disrupted in ONFH [13], and bone mass inversely correlates with fat mass in the bone marrow microenvironment [14]. Several studies suggest that the mechanisms of ONFH may involve alcohol and glucocorticoids (GCs) directly promoting adipogenesis while inhibiting osteogenesis in MSCs [11, 15–17]. As such, suppressing marrow adipogenesis may be a therapeutic strategy to prevent increased formation of adipocytes and promote an increase in functional bone cells [18], potentially favoring the bone marrow microenvironment. The bone marrow microenvironment (BMM) is a complicated cellular and molecular system composed of MSCs, endothelial cells, nerves from the sympathetic nervous system, accessory cells (T lymphocytes and monocytes), etc., which act interactively and play a role in bone marrow homeostasis [19].

Bone marrow adipose tissue (BMAT), accounting for approximately 70% of the bone marrow volume in adult humans, was discovered over a century ago [20]. Traditionally, adipose tissue (AT) is segregated into white AT (WAT), which is responsible for endocrine functions, energy storage and release, and brown AT (BAT), which controls adaptive thermogenesis in mammals. The stimulation from cold exposure can transform WAT into beige adipocytes, also known as brown-like adipocytes [21]. BMAT, which has microstructural similarities to WAT, is a distinct type of adipose tissue. This tissue has a unique molecular composition, setting it apart from WAT and BAT [22]. In addition, under pathological conditions, marrow adipogenesis-triggered intraosseous hypertension is believed to compress venous sinusoids, leading to intravascular coagulation and blocking arterial blood flow [23]. In contrast, fat tissue has been identified as a critical endocrine organ that secretes numerous hormones and adipokines. Recent reports have shown that visceral adipocytes secrete various physiologically active substances known as adipokines [24, 25]. Among these substances, more than 50 adipokines, ranging from cytokines and growth factors, are known to be alternative complement system proteins and exert major effects on metabolism, inflammation, and angiogenesis [26]. Various studies, including ours, have explored the impacts of various adipokines on the skeletal system. For example, bone marrow adipocytes express RANKL and promote osteoclast differentiation [27, 28], contributing to subchondral bone destruction [29]; elevated adiponectin levels increase fracture risk, which could be attributed to its ability to reduce bone mass [24]; strong PAI-1 expression in bone marrow adipocytes could lead to intravenous thrombus formation via a paracrine interaction [25]; and the anabolic effect of leptin promotes MSC differentiation into osteoblasts but suppresses adipogenesis [26]. Thus, adipocytes and various adipokines likely play crucial roles during the process of ONFH development [30].

To date, the use of BMSCs has been limited in regenerative medicine for the treatment of multiple musculoskeletal problems, such as osteonecrosis, osteoarthritis and bony defects [31]. More recently, the implantation of adipose-derived mesenchymal stem cells (ADMSCs) has become a promising method for stem cell therapy because of the multimesenchymal lineage potential of these cells, which can differentiate into osteoprogenitor cells under appropriate conditions. Additionally, the ease of harvesting ADMSCs and the possibility of expanding cell lines make sufficient cell numbers highly achievable [32]. Therefore, in this review, we describe the pathomolecular mechanisms of ONFH, with a focus on BMSCs, ADMSCs, and adipocytes. We analyze and interpret associated cytokines and signaling pathways, as well as recent treatment modalities and their efficacy, aiming to shed light on and inspire future therapeutic approaches for ONFH.

## Changes in the balance between the osteogenesis and adipogenesis of BMSCs in ONFH

### Normal differentiation of BMSCs

BMSCs, a prevalent type of stem cell, can multiply and differentiate into osteoblasts, adipocytes, fibroblasts, chondrocytes, and neurocytes [33]. However, an increase in adipogenesis suppresses osteogenesis in BMSCs, as these two processes are competitively balanced [15, 34]. Both marrow adipocytes and osteoblasts originate from BMSCs, and the determination of their cellular destiny is governed by finely regulated lineage-specific transcription factors [35]. Under physiological conditions, BMSCs derived from humans powerfully differentiated into osteoblasts, with significantly increased expression of alkaline phosphatase (ALP), Runt-related transcription factor 2 (Runx2), Collagen 1 (COL 1), and osteocalcin (OC) in vitro [36]. Moreover, BMSCs possess immunomodulatory properties without immunogenicity, making them ideal candidates for repairing tissue damage and treating inflammatory diseases [37–39].

### An imbalance in the osteogenesis and adipogenesis of BMSCs leads to ONFH

**Fig. 1:**
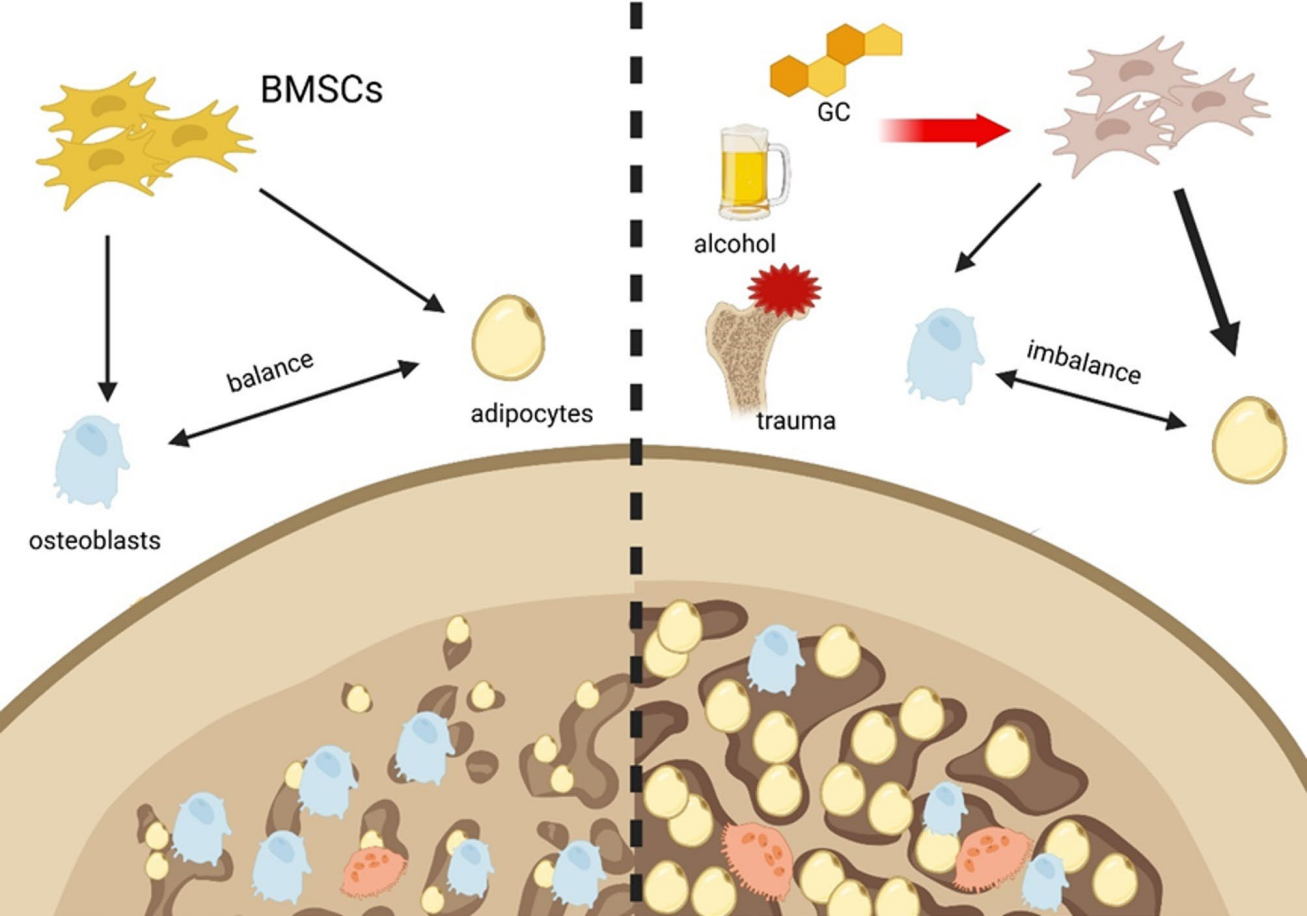
An imbalance between the osteogenic and adipogenic differentiation of BMSCs leads to ONFH. Under physiological conditions, the osteogenic and adipogenic differentiation of BMSCs is in competitive equilibrium. However, factors such as trauma, alcohol, and glucocorticoids directly promote adipogenesis while simultaneously inhibiting osteogenesis in MSCs. This shift toward diminished osteogenic differentiation and increased adipocytic differentiation of BMSCs is a contributing factor to the development of ONFH

Marrow adipocytes and osteoblasts primarily originate from BMSCs, whose differentiation is closely related. When differentiation toward the adipocytic phenotype occurs, it detrimentally affects the osteoblast phenotype [18]. The diminished ability for osteogenic differentiation and increased capability for adipocytic differentiation of BMSCs are implicated in the development of ONFH [13]. In turn, adipocytic differentiation of BMSCs increases fatty accumulation and leads to elevated intraosseous pressure in the femoral head. This pressure eventually obstructs blood circulation, thus promoting ONFH progression [9, 23] (Fig. 1). Multiple signaling pathways and cytokines are involved in the balance between osteoblastic and adipocytic differentiation in BMSCs but lead to a switch in the expression of osteogenic markers (Adipoq and PPAR-γ) and adipocytic markers (ALP, RUNX2 and OCN) [13].

### Key factors regulating the balance of the adipocytic and osteogenic differentiation of BMSCs

#### RUNX2

Runx2, also known as Core Binding Factor Alpha 1, is an important transcription factor necessary for both the early and late stages of osteoblastic differentiation. This molecule directs mesenchymal progenitors toward the osteoblast lineage [40]. The role of Runx2 in osteogenesis was elucidated through experiments in mice with homozygous Runx2 mutations. These Runx2−/− mice die shortly after birth and exhibit incomplete ossification of their skeleton [38, 41]. Runx2 expression is substantially reduced in the bone tissues of patients with steroid-induced ONFH and appears to influence various therapeutic signaling pathways, such as the Wnt/β-catenin signaling pathway, BMP superfamily, and PI3K/AKT pathway [17, 42–44]. GC-induced osteogenesis is negatively regulated by Runx2/Cbfal serine phosphorylation [3]. The activation of Runx2 in BMSCs can expedite osteoblast differentiation and substantially attenuate adipocytic differentiation. Furthermore, exogenous Runx2 can counteract the dexamethasone (DEX)-mediated promotion of adipocytic differentiation. These observations suggest that a precisely regulated increase in Runx2 expression or its downstream effectors might mitigate the severe side effects of long-term GC therapy [40]. Overexpressing P-glycoprotein increases Runx2 expression and ALP activity, suggesting that P-glycoprotein promotes DEX-induced osteogenesis in BMSCs. PPARγ can suppress the expression of Runx2, thereby inhibiting osteogenesis [15].

#### PPARγ and C/EBPs

PPARγ, an adipocytic transcription factor, belongs to the nuclear hormone receptor subgroup, and its activity is regulated by ligands. The development of ONFH is strongly correlated with increased PPARγ expression. Furthermore, the downregulation or suppression of PPARγ in BMSCs may inhibit steroid-induced adipocytic differentiation, thus providing a possible prevention method for ONFH [43]. Research suggests that steroids stimulate PPARγ gene expression and that alcohol can increase PPARγ mRNA expression in BMSCs, as evidenced by in vivo and in vitro studies [3, 16]. As exemplified by 3T3-L1 and 3T3-F442A cells, PPARγ mRNA often shows signs of activation before other adipocyte genes during adipocytic differentiation [45]. Xu HH et al. revealed that platelet-rich plasma markedly reduced the expression levels of PPAR-γ and decreased the serum triglyceride (TG) and total cholesterol (TC) levels [1]. Emerging evidence shows that MFAP5 and SND1 are upstream molecules of the PPARγ signaling pathway. MFAP5 was found to directly bind to and inhibit the expression of SND1. As a novel coactivator of peroxisome PPARγ, SND1 is involved in the MFAP5-mediated negative regulation of adipocytic differentiation [46]. Studies have demonstrated the importance of the C/EBP transcription factor family (C/EBPα, β, δ) in adipogenesis. These molecules participate in regulating adipocytic genes and affect the uptake of glucose by adipocytes. While C/EBPβ and C/EBPδ initiate lipogenic signals at the early stage of lipid differentiation and then decrease rapidly afterward, C/EBPα remains consistently present. Specifically, the transcription factor C/EBPα could directly promote the transcriptional activity of the PPARγ promoter region. The importance of C/EBPα and PPARγ in adipocytic differentiation has been confirmed in numerous studies [11].

#### MicroRNAs

MicroRNAs (miRNAs) are small, single-strand noncoding RNA molecules that are typically composed of approximately 22 nucleotides. These molecules are important for bone remodeling and play critical roles in the pathogenesis and treatment of ONFH [47, 48]. Below are the most studied ONFH-associated miRNAs (Table 1).

**Table 1:** RNAs in the pathogenesis and treatment of ONFH

Name	Effect on BMSC	Reference
MiRNA-320a	targets RUNX2 and inhibits the osteoblast differentiation of BMSCs	[48]
MiR-93-5p	inhibits osteoblast differentiation via BMP-2	[4]
MiR-224-5p	suppresses osteogenic but promotes adipogenic differentiation of BMSCs via Smad4 and TAZ	[49]
MiR-100-5p	suppresses the osteogenic differentiation of BMSCs by inactivating the BMPR2/Smad1/5/9 signaling pathway	[50]
MiR-27a	promotes osteogenic differentiation of BMSCs by targeting the PI3K/Akt/mTOR pathway	[42]
MiR-204-5p	inhibits osteogenic differentiation by targeting Runx2	[54]
MiR-125a-3p	inhibits osteogenic differentiation by targeting GILZ	[54]
TCONS_00041960	promotes osteogenic differentiation by directly sponging miR-204-5p and miR-125a-3p	[54]
MiR-30a	inhibits osteogenic differentiation	[55]
LncRNA RP11-154D6	promotes osteogenic differentiation by interacting with miR-30a	[55]
HOTAIR	inhibits osteogenic differentiation	[56]
CircRNA CDR1	inhibits osteogenic differentiation	[51]
MiR-34c-5p	promotes adipogenic differentiation	[69]

Patients with trauma-induced osteonecrosis of the femoral head (TIONFH) exhibit high expression of miR-93-5p in the peripheral blood during disease. This oncomiR likely impedes osteoblast differentiation via BMP-2, leading to a substantial decrease in ALP and the formation of calcium nodules. Notably, miR-93-5p has been found to promote the proliferation of hBMSCs in vitro [4]. Notably, miRNA-320a expression was upregulated, and Runx2 expression was significantly downregulated in patients with TIONFH. Moreover, the overexpression of miRNA-320a inhibited the expression of osteogenesis-associated proteins, including Runx2, Collagen I and OCN. This overexpression also hampers the osteogenesis of BMSCs [48].

MiR-224-5p is upregulated in GC-treated BMSCs and has been found to suppress osteogenesis while promoting the adipocytic differentiation of BMSCs. Additionally, Smad4, a crucial conductor of bone morphogenetic protein and an important component of TGF-beta signaling, was predicted to be a target gene of miR-224-5p. Furthermore, Smad4, together with TAZ, is important for regulating cell fate and maintaining stem cell self-renewal. GC-induced upregulation of miR-224-5p potentially mediates the adipo-osteogenic differentiation of BMSCs through Smad4 targeting. The miR-224-5p-Smad4-TAZ axis might be a potent signaling mechanism, and the inhibition of miR-224-5p expression could promote osteogenesis and reduce adipogenesis, hence alleviating the occurrence of ONFH. This discovery suggests a novel therapeutic target for the treatment of steroid-induced ONFH (SIONFH) [49].

Exosomes, which act as new mediators for intercellular signal transmission, have been reported to be closely linked to many bone and joint diseases, including osteoarthritis, rotator cuff injury, and osteoporosis. MiR-100-5p expression was found to be upregulated in ONFH exosomes, and this upregulation suppressed the differentiation of hBMSCs by targeting BMPR2 and deactivating the BMPR2/Smad1/5/9 signaling pathway [50].

MiR-27a plays an essential role in the osteoprotection of SIONFH patients by regulating the osteogenic differentiation of BMSCs. This oncomiR has been found to increase ALP activity and the expression of the osteogenesis-related marker Runx2 while downregulating the expression of the adipogenic marker PPARγ [42].

#### Circular RNAs

Circular RNAs (circRNAs) are a type of covalently closed noncoding RNA, and recent evidence indicates that they can influence gene expression via various mechanisms, thereby regulating the occurrence and development of diseases [51]. The advancements in emergent sequencing technology and bioinformatic analysis techniques have shifted the understanding of circRNAs from mere splicing byproducts to a new focal point in ONFH research. CircRNAs can bind miRNAs and function through a competing endogenous RNA (ceRNA) mechanism. The downregulation of hsa_circ_0000219 and hsa_circ_0005936 may contribute to ONFH progression primarily by affecting the differentiation of BMSCs through the hsa_circ_0000219‑miR‑144‑3p or hsa_circ_0005936‑miR‑1270 axis. Upregulated circRNAs may be potential tools for rescuing the impaired proliferation and osteogenic capacity of BMSCs and promoting bone repair in the necrotic area in ONFH. However, further studies are needed to elucidate the exact functions of circRNAs and to evaluate their potential for early interference in ONFH patients [52].

#### Long noncoding RNAs

Long noncoding RNAs (lncRNAs) represent a newly discovered category of regulatory molecules implicated in various biological processes, including cell differentiation and gene expression regulation. Operating as ceRNAs, lncRNAs compete for microRNA binding to govern gene expression. Studies have revealed the regulatory role of lncRNAs in the osteogenic differentiation of BMSCs [53]. For example, TCONS_00041960 functions as a ceRNA by directly sponging miR-204-5p and miR-125a-3p, thereby promoting the expression of Runx2 and inhibiting the expression of GILZ in GC-treated BMSCs, respectively. Consequently, the novel TCONS_00041960-miR-204-5p/miR-125a-3p-Runx2/GILZ axis participates in the adipogenesis and osteogenesis of these GC-treated BMSCs [54].

Moreover, decreased expression of the lncRNA RP11-154D6 has been reported in the BMSCs of patients with ONFH. Overexpressing the lncRNA RP11-154D6 promoted transdifferentiation of the cells toward an osteogenic route and suppressed their contribution to adipogenesis. Thus, downregulation of the lncRNA RP11-154D6 may augment lipid accumulation and curtail bone defect repair, suggesting that the lncRNA RP11-154D6 operates by interacting with miR-30a, a microRNA known for its inhibitory role during the osteogenic differentiation of BMSCs. The lack of a significant relationship between the expression levels of lncRNA RP11-154D6 and miR-30a-5p indicates that lncRNA RP11-154D6 might regulate differentiation through mechanisms other than merely targeting miR-30a-5p [55].

HOTAIR, a well-characterized oncogenic lncRNA, has been shown to inhibit BMSC osteogenic differentiation in nontraumatic osteonecrosis of the femoral head. Neohesperidin, a natural flavanone glycoside utilized as an herbal medicine in China and recognized for its diverse pharmacological properties, has been found to ameliorate SIONFH by inhibiting the histone modifications of the lncRNA HOTAIR [56].

#### Other regulators controlling the balance between the osteogenesis and adipogenesis of MSCs

Among the regulators that control the balance between the osteogenesis and adipogenesis of MSCs, RNA methylation substantially contributes, accounting for more than 60% of RNA modifications. The most prevalent RNA methylation in eukaryotic cells is m6A. A methyltransferase complex, which includes m6A writers such as METTL3, METTL14 and WTAP, catalyzes the formation of m6A methylation. Notably, in necrotic bone tissues and SONFH BMSCs, the expression level of METTL14 was reduced, leading to a decrease in the m6A level. Moreover, by increasing the m6A level of PTPN6 (a protein tyrosine phosphatase), the m6A methyltransferase METTL14 can promote the proliferation and osteogenic differentiation of SONFH BMSCs by activating the Wnt signaling pathway. Therefore, these findings suggest that the METTL14‒PTPN6 axis may be a potential therapeutic target for SIONFH [57].

### Major signaling pathways involved in the adipocytic and osteogenic differentiation of MSCs

#### Wnt/β-catenin signaling

The Wnts (Wnt-1, Wnt-3a, Wnt-5b, Wnt-7a, and Wnt-10b) interact with membrane-bound frizzled receptors and the LRP5/6 coreceptor, initiating a signaling cascade and leading to the nuclear aggregation of β-catenin. This binding prevents the cytoplasmic degradation of β-catenin and stimulates its nuclear translocation. Inside the nucleus, β-catenin binds to and coactivates the T-cell factor/lymphoid-enhancing factor family of transcription factors [58, 59]. Previous reports showed that Wnt/β-catenin signaling can inhibit the early stages of adipocyte differentiation [60] and promote osteoblast differentiation [38].

Steroid treatment affects the Wnt signaling pathway, reducing bone formation by inhibiting the activity of β-catenin and modulating the expression of Wnt signaling-related molecules in osteoblasts. Pravastatin may prevent SIONFH by suppressing PPARγ expression and activating the Wnt signaling pathway. These findings highlight the regulatory impact of pravastatin on adipogenesis and osteogenesis during the progression of SIONFH [43]. The Wnt/β-catenin pathway plays a pivotal role in the osteogenesis of BMSCs. Yu et al. recently reported that the level of β-catenin was markedly reduced by ethanol. However, osthole can reinstate β-catenin in a dose-dependent manner, augmenting osteogenesis [5]. A recent report revealed significant inhibition of β-catenin signaling in SIONFH patients and corresponding rat models, whereas the administration of the Wnt agonist 1 attenuated the accumulation of fat droplets and sparse trabeculae in SIONFH model rats via the activation of β-catenin signaling [61]. Moreover, previous studies have indicated that glycyrrhizic acid mediates its effects by activating the Wnt/β-catenin pathway, which reduces the oxidative stress levels induced by excessive GCs. Consequently, this change increases osteogenic differentiation, attenuates lipogenic differentiation of MSCs, and ultimately maintains osteolipogenic homeostasis [12].

Interestingly, there is mutual similarity between the risk factors for ONFH and the factors leading to an increase in serum amyloid A (SAA) levels. Regardless of whether alcohol, hormones, or trauma triggers an increase in SAA expression, the molecular mechanism underlying this phenomenon shows that SAA can inhibit Wnt/β-catenin signaling and activate the downstream PPARγ of the MAPK signaling pathway [7]. Silencing of the cryptochrome gene 1 leads to increases in both total β-catenin and nuclear β-catenin, coupled with decreases in GSK-3β under adipogenic stimulation. These results demonstrate that downregulating CRY1 may initiate the canonical Wnt/β-catenin signaling pathway. Intriguingly, one study revealed that CRY1 can act as a novel adipogenic differentiation regulator in vitro; knockdown of this gene may inhibit adipogenic differentiation, partially through the canonical Wnt/β-catenin signaling pathway [62]. Further gene knockout studies utilizing advanced CRISPR/Cas9 editing technology are expected [63].

Moreover, the circRNA CDR1 affects the osteogenic and adipogenic differentiation of BMSCs via the miR-7-5p/WNT5B pathway. An increase in the expression of CDR1as in SIONFH-BMSCs promotes WNT5B expression by competitively binding to miR-7-5p. Elevated WNT5B expression subsequently inhibits the expression of β-catenin, a prominent player in the WNT/β-catenin signaling pathway, which was negatively correlated with the expression of WNT5B in Gaoyang Chen’s study [51]. As a Wnt signaling receptor, Frizzled1 (FZD1) plays an important role in osteoblast mineralization. Several transcription factors, including Sp1, E2F1, and AP2, are involved in the regulation of the FZD1 promoter. Previous reports showed that aberrant CpG island hypermethylation of the FZD1 gene is present in patients with ONFH, resulting in Wnt/β-catenin signaling inactivation and subsequent cell dysfunction. By comparative observation, another study confirmed that at an appropriate concentration, 5’-Aza-dC (an inhibitor of DNA methyltransferase) benefits MSCs in patients with GC-associated ONFH by inducing de novo FZD1 expression [64].

#### TGFβ/BMP superfamily

Bone morphogenetic protein (BMP), an endogenous mediator, is integral to fracture repair. As an acidic glycoprotein, it is synthesized and secreted predominantly from osteoblasts and is widely present in the bone matrix. Currently, there are more than 20 BMP family members, a robust group of osteogenic factors that can stimulate bone mesenchymal progenitor cells to differentiate into mature osteoblasts. BMP2, the most pivotal extracellular signaling molecule, promotes bone formation, induces bone cell differentiation, and regulates the gene expression of a variety of osteogenic transcription factors, such as Osterix and Runx2 [44].

Bone morphogenetic protein receptor 2 (BMPR2) can directly phosphorylate and activate BMPR1, which then phosphorylates SMAD1/5/9 and promotes the cohesion and nuclear translocation of SMAD1/5/9 and SMAD4, thus promoting the activation of osteogenesis and angiogenesis while inhibiting adipogenesis [50]. The Smad proteins play important roles in the TGF-β/BMP signaling pathway. Prior studies have shown that Ski can interact with Smads and suppress BMP signaling. Moreover, Ski acts as a negative regulator of TGF-β signaling via interactions with Smad proteins. In terms of this point, any factors leading to a surge in Ski (a multifunctional transcriptional regulator) can negatively regulate the TGF-β/BMP-2 signaling pathway. Additionally, Xin Zhao reported that Ski was significantly increased in the SIONFH model, mirroring the expression of the adipogenic proteins PPAR-γ and FABP4. Moreover, Ski knockdown reduced the adipogenic differentiation of BMSCs, suggesting that, as a novel molecule, Ski may regulate adipogenesis processes in the pathological conditions of SIONFH [65]. SIRT2, a classic NAD+-dependent deacetylase, serves as an upstream suppressor of BMP2. The knockdown of SIRT2 notably elevates the expression levels of BMP2, thereby promoting the osteogenic differentiation of BMSCs and mitigating GC-induced oxidative stress and cell apoptosis [66].

#### PI3K/AKT pathway

**Fig. 2:**
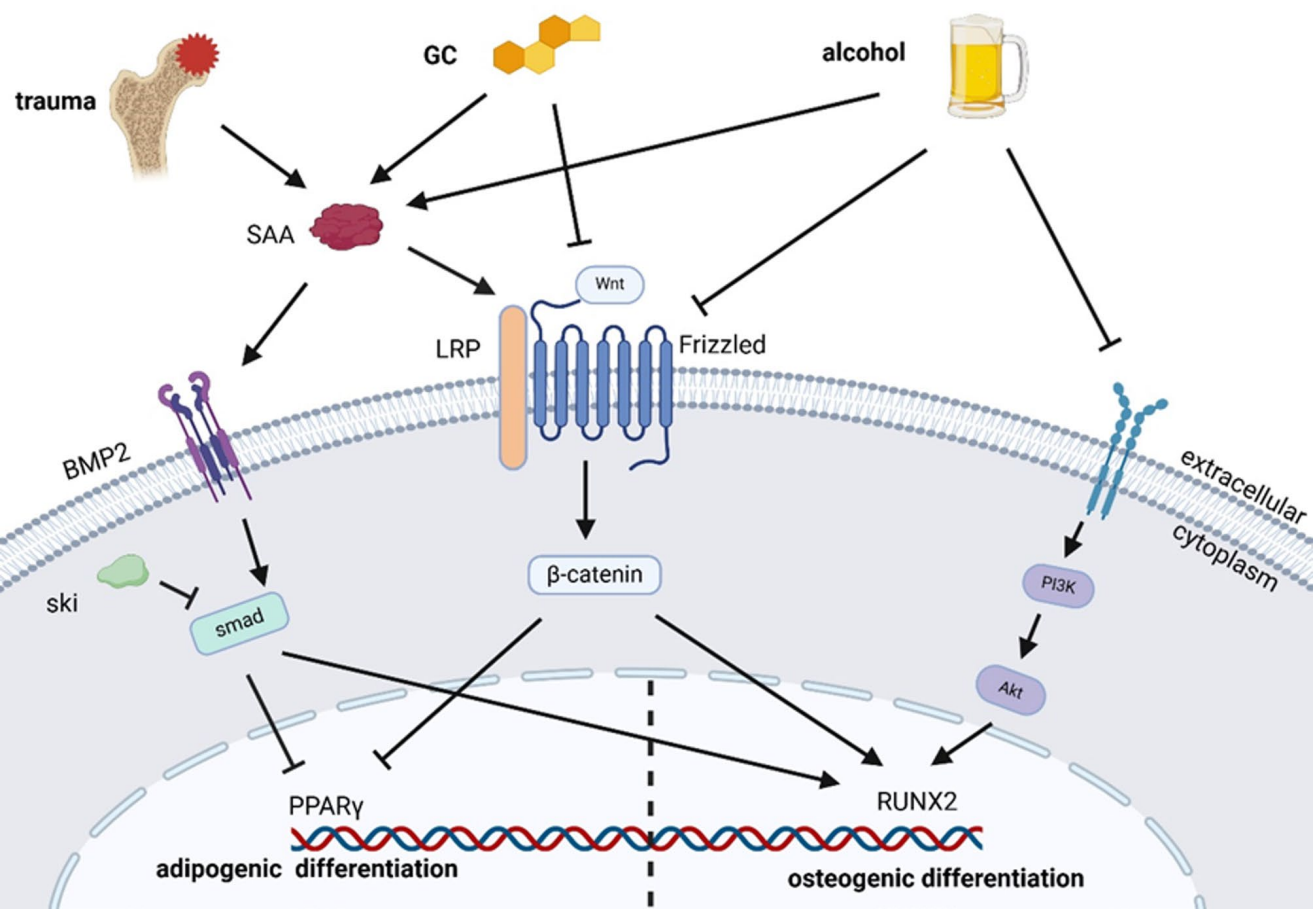
Major signaling pathways and transcription factors in BMSCs. Factors such as alcohol, hormones, or trauma trigger an increase in SAA expression. SAA inhibits the Wnt/β-catenin signaling pathway and activates the downstream PPARγ of the MAPK signaling pathway. Stimulations with GCs and alcohol can directly reduce bone formation by inhibiting the activity of β-catenin by impeding the Wnt signaling pathway. BMP2 can phosphorylate Smad1/5/9 and drive the activation of osteogenesis and angiogenesis while inhibiting adipogenesis. However, Ski, which functions as a negative regulator through interactions with Smad proteins, can promote adipogenic differentiation. The PI3K/AKT pathway, which is influenced by alcohol stimulation, inhibits proliferation and osteogenic differentiation. These signaling pathways target crucial transcription factors such as Runx2 for osteogenesis and PPARγ and C/EBPs for adipogenesis

Many studies have suggested that the PI3K/AKT pathway is a key regulator of the osteogenesis of BMSCs. This pathway is involved in the osteogenic differentiation associated with alcohol-induced bone loss. In vitro studies revealed that alcohol significantly inhibited the proliferation and osteogenic differentiation of BMSCs but stimulated adipogenic differentiation. However, chrysophanic acid (CPA) can partially counteract the negative impact on osteogenesis via the PI3K/AKT pathway [17]. A recent study confirmed that monoacylglycerol lipase (MAGL) participates in the regulation of BMSC fate and that MAGL inhibition can effectively reverse the effect of GCs on BMSC differentiation by activating the PI3K/AKT/GSK3β signaling pathway [67]. Moreover, another study indicated that Alda-1 (a highly selective agonist for aldehyde dehydrogenase 1) can alleviate the inhibitory effect of ethanol on the osteogenesis of BMSCs via PI3K/AKT signaling. Therefore, PI3K/AKT signaling appears to be a critical pathway for the osteogenic differentiation of BMSCs [68] (Fig. 2).

#### The miR-34c-5p/MDM4 pathway

MiR-34c-5p is highly expressed in the femoral heads of patients with SIONFH. There was a significant increase in the expression of miR-34c-5p in the DEX-induced BMSCs, and the overexpression of miR-34c-5p promoted adipogenic differentiation in the BMSCs. This overexpression has been confirmed to play pivotal roles in GC-induced adipogenic differentiation, and MDM4 was identified as the downstream target gene. MDM4 serves as a direct target of miR-34c-5p and is negatively regulated by it. ShRNAs posttranscriptionally downregulate the expression of MDM4 to promote lipid droplet accumulation in BMSCs [69].

**Fig. 3:**
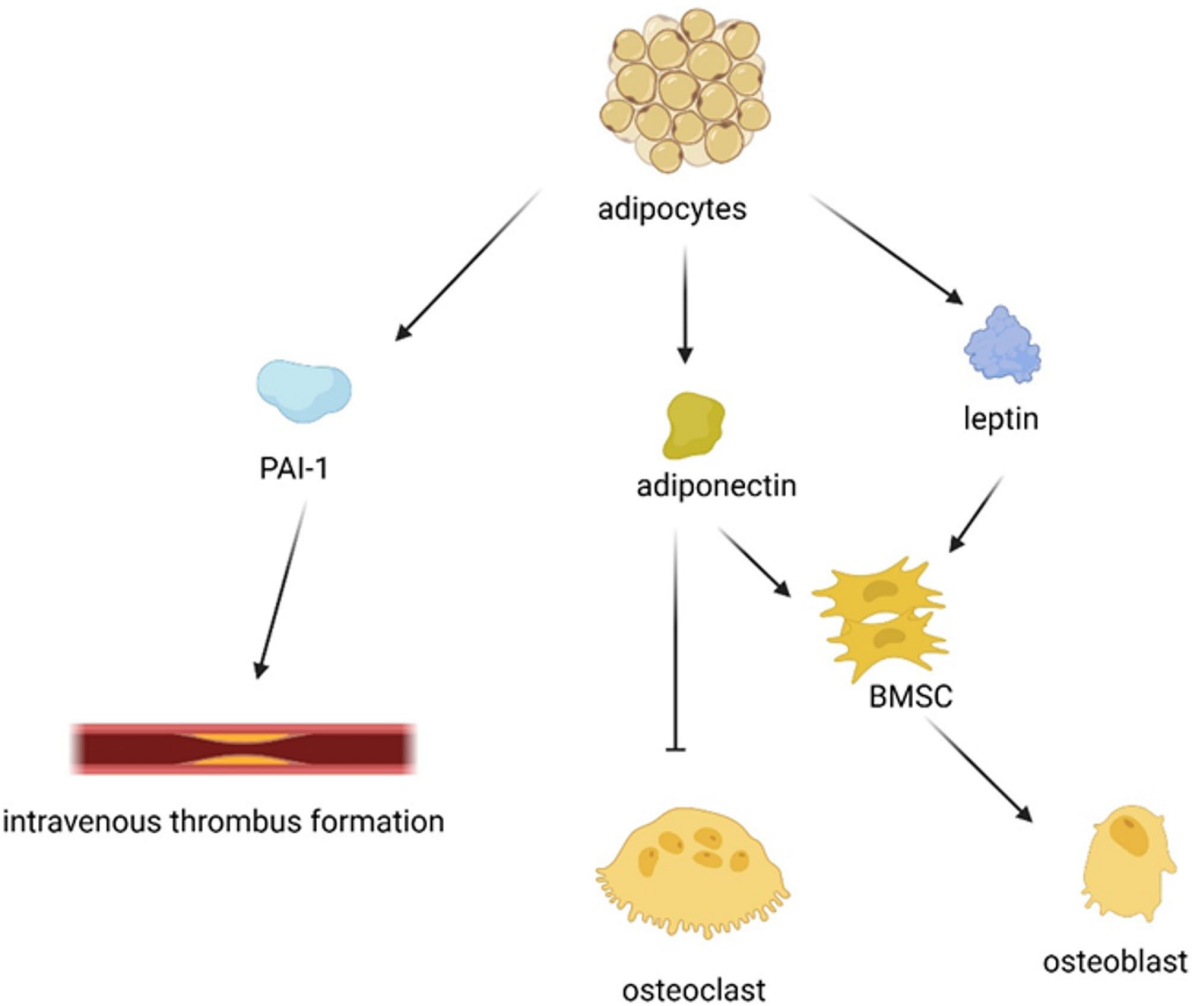
Adipocytes emit a variety of adipokines that participate in the process of femoral head necrosis. PAI-1, an essential extravenous factor, is involved in intravenous thrombus formation through paracrine cell-to-cell interactions. Adiponectin has the potential to increase bone mass by suppressing osteoclastogenesis and stimulating osteoblastogenesis. Furthermore, leptin aids in the differentiation of BMSCs into osteoblasts

## Adipocytes release a variety of adipokines involved in the process of femoral head necrosis

### The origin and endocrine function of adipose cells in the femoral head

Research suggests that visceral adipocytes secrete various physiologically active substances, known as adipokines. The bone marrow space is rich in mature adipocytes, which are potential candidates for adipokine secretion [24] (Fig. 3).

BMAT could be an integral component of the bone marrow microenvironment [70]. As a major endocrine organ, BMAT releases numerous hormones and adipokines, which have diverse systemic effects. Moreover, the secretion of adiponectin from BMAT is considerably greater than that from WAT in humans. Owing to this adiponectin production, BMAT may influence systemic effects on bone homeostasis, immune responses, vascular activities, and cancer risk [20, 24]. Certain adipocytes within BMAT exhibit features akin to brown adipocytes when subjected to environmental or drug stimuli such as exercise, cold exposure, or medication. These cells, termed beige (brown-in-white) adipocytes, lead to a process referred to as “browning” [71]. BAT thermogenesis relies on an abundance of mitochondria and the high expression of uncoupling protein 1 (UCP-1), which is located on the mitochondrial inner membrane. This molecule catalyzes the uncoupling of fuel combustion (proton leakage) from ATP production, leading to the expenditure of energy as heat. Thus, methodologies that activate brown adipocytes could have major health implications, especially potent antiobesity and antidiabetic properties [72]. However, a direct link between BAT and bone homeostasis within the bone marrow microenvironment has yet to be established.

Plasminogen activator inhibitor-1 (PAI-1), an adipokine, suppresses fibrinolysis by binding tissue-type plasminogen activator (t-PA). This interaction implies an association between PAI-1 and thrombosis or hypercoagulation [24]. Indeed, significant PAI-1 expression in bone marrow adipocytes may be an important extravenous factor that induces intravenous thrombus formation via paracrine cell–cell interactions. The production of PAI-1 in bone marrow adipocytes induced by dexamethasone (DEX) could be one of the pathogeneses of osteonecrosis [25].

Adiponectin, known for its antiatherogenic and anti-inflammatory properties, is involved in the regulation of lipid metabolism. Abnormal lipid metabolism and bone marrow fat cell hypertrophy/proliferation are important contributors to nontraumatic ONFH. Studies also link adiponectin to bone metabolic regulation, suggesting that it can suppress osteoclastogenesis and activate osteoblastogenesis [73]. However, adiponectin can also negatively impact bone mass, increasing the risk of bone fracture [24]. In a clinical trial, patients with nontraumatic ONFH had notably lower plasma adiponectin levels than healthy controls did, as did patients with traumatic ONFH or hip OA [30].

Leptin, known for its anabolic effect on osteoblasts, promotes the differentiation of MSCs into osteoblasts while limiting adipogenesis. Leptin inhibits receptor activator of nuclear factor kB ligand (RANKL) production and increases osteoprotegerin production, resulting in restricted osteoclast development [74]. An increase in leptin expression could diminish the anabolic effect of leptin on adipocytes, leading to increased osteoblast production. Leptin can trigger JAK3/STAT3 activation and subsequently inhibit adipogenesis [75].

Simvastatin decreases the mRNA expression and protein secretion of PAI-1 in human bone marrow adipocytes, also suppressing DEX-induced PAI-1 secretion. These findings may reveal one of the mechanisms by which simvastatin may prevent SIONFH [27]. The present in vitro study demonstrated that the phytoestrogenic molecule desmethylicaritin, a unique metabolite of *Epimedium*-derived flavonoids, inhibits adipogenesis by downregulating the expression of the adipogenic transcription factors C/EBPα and PPARγ. Wnt/β-catenin signaling may be regulated by desmethylicaritin during its suppression of adipogenesis, with Wnt10b identified as a key factor in inhibiting adipogenesis in Wnt signaling [76].

## Emerging applications of adipose-derived mesenchymal stem cells in osteonecrosis of the femoral head

The implantation of adipose-derived mesenchymal stem cells (ADMSCs) has recently emerged as a viable approach for stem cell therapy given the multi-mesenchymal lineage potential of these cells and their ability to differentiate into osteoprogenitor cells under appropriate conditions. While ADMSCs have a natural tendency to differentiate into adipocytes in vitro, ADMSCs have unique advantages over BMSCs in stem cell therapy, such as high quantities, easy acquisition, and increased proliferation [77, 78]. These findings suggest that ADMSC implantation is a promising direction for the treatment of ONFH. Moreover, several studies have explored the efficacy of ADMSC implantation for ONFH therapy. A recent finding indicated that culture-expanded ADMSC implantation is not only safe but also has a minimal risk of systemic or surgical adverse events [32].

ADMSCs subjected to manual intervention demonstrated superior osteogenic potential, and ADMSC implantation could be a promising direction in the treatment of ONFH. Furthermore, another study showed that dimethyloxaloylglycine (DMOG)-treated adipose‑derived stem cells (ASCs) significantly increased vascularization and bone regeneration in the necrotic area of the femoral head in ONFH model rabbits. These findings suggest that DMOG can amplify the osteogenic activity of ASCs in vivo and therefore represents a novel and effective therapeutic intervention for early-stage corticosteroid-induced ONFH [79]. Previous studies have pioneered BMP2/VEGF-transfected ASC therapeutics to simultaneously promote osteogenesis and angiogenesis. The optimal ratio of BMP2- to VEGF-transfected ASCs was determined to be 9:1. As a result, an increase in the expression of BMP2 and VEGF in ASCs could alter Hippo pathway dynamics, thereby increasing bone differentiation and neovascularization. Moreover, the elevation in TAZ promoted osteogenic differentiation with Runx2 and, in conjunction with elevated TEAD1, improved cell proliferation and angiogenesis via an increase in ANKRD1 expression [80].

However, chronic treatment with GCs compromises the bone regenerative capacity and osteogenic capability of ADMSCs due to a significant increase in Dkk-1 expression, a factor that counters Wnt/β-catenin signaling. Intriguingly, ADMSCs exposed to steroids exhibit a proliferative ability akin to that of regular ADMSCs [81]. A study also revealed that miR-378-ASC-Exos bolstered the osteogenic differentiation of BMSCs amidst the negative impact of GCs. These findings suggest that miR-378-ASC-Exos promote osteogenesis and alleviate GC-induced ONFH. Moreover, miR-378 can be transferred into recipient cells via exosomes, resulting in the downregulation of Sufu and subsequent activation of the Shh signaling pathway. This process could potentiate neovascularization and osteogenesis through the activation of the Shh signaling pathway [47].

## Emerging regenerative stem cell therapy in ONFH

### Potential roles of extracellular vesicles

The role of MSCs in the progression of ONFH has increasingly been acknowledged, leading to interest in regenerative therapeutics for disease alleviation. Studies with a rat model of SIONFH demonstrated significant alleviation of bone tissue necrosis following the injection of exosomes derived from human umbilical cord MSCs. This improvement manifested primarily as reduced apoptosis of bone tissues, increased trabecular reconstruction, and increased angiogenesis in necrotic bone tissues [82]. Growing evidence has revealed that Exos derived from BMSCs reduce the formation of lipid droplets, thereby promoting osteogenesis, and may serve as an immunotherapeutic strategy for SIONFH [83]. BMSC-derived exosomes, which contain miR-668-3p, promote osteoblast progression in ONFH by upregulating the expression of CD63 and CD9 [84]. In addition, BMSC-derived exosomes carry miR-122-5p and promote osteoblast proliferation, differentiation, and angiogenesis in rabbit models of ONFH [85]. Recent research has indicated that extracellular vesicles (EVs) from osteogenically differentiated human BMSCs increase the viability and reduce the degree of apoptosis of native hBMSCs. Thus, Exo treatment could serve as a promising, noncellular, and nondrug-mediated approach to promote bone health. Osteogenic Exos positively affect the osteogenic differentiation capacity of precursor cells but inversely affect the adipogenic differentiation capacity of hBMSCs [86].

### Pharmacologic interventions targeting MSC function

Various drugs may be attractive candidates as potential pharmacotherapeutic agents for early-stage ONFH due to their protective properties. The saturated fatty acid (SFA) palmitic acid (Palm; C16:0) has been reported to induce apoptosis in human MSCs and osteoblasts. Moreover, the potential protective role of stearoyl-CoA 9-desaturase 1 (SCD1), an enzyme responsible for the desaturation of SFAs to monounsaturated fatty acids (MUFAs), against the deleterious effects of Palm has been investigated. Its activation could alter the intracellular SFA/MUFA ratio, positioning SCD1 as a protector against lipotoxicity and indicating it has an essential role in BMSCs [87]. A recent study reported that valproic acid, a commonly used antiepileptic and anticonvulsant drug, increased the osteogenic differentiation of BMSCs while attenuating the deleterious effects of GCs on BMSC proliferation, apoptosis and osteogenic differentiation. Consequently, VPA may serve as a potential pharmaceutical intervention for preventing ONFH, possibly via the increase in the osteogenic differentiation of BMSCs [88]. Notably, polydatin (PD) augmented the proliferation and osteogenic differentiation of hBMSCs by activating the BMP2-induced Wnt signaling pathway and inducing the accumulation and nuclear translocation of β-catenin [89]. For the first time, a study revealed that Jintiange capsules can promote osteogenesis and inhibit adipogenesis by affecting the activity of BMSCs. This effect is attributed to the increased level of β-catenin, potentially halting the progression of early-stage SIONFH [90]. In another study, lithium was reported to bolster the osteogenic function of BMSCs, primarily via the increase in exosomal Wnt10a secretion, causing β-catenin activation, a previously undisclosed mechanism. GelMA hydrogels have been employed to facilitate the sustained release of Li-Exos, thereby promoting bone repair in vivo [91].

### Combined application of functionalized biomaterial scaffolds and MSCs

Composite implants of carboxymethyl chitosan/alginate/bone marrow mesenchymal stem cell/endothelial progenitor cell (CMC/ALG/BMSC/EPC) appear to promote the repair of SIONFH by promoting osteogenesis and angiogenesis, coupled with the attenuation of adipogenesis. Thus, when accompanied by core decompression, the cotransplantation of BMSCs and EPCs onto 3D scaffolds might be a feasible therapeutic strategy for SIONFH [92]. Moreover, the use of a single injection of 3D microscaffolds endowed with low-dose BMSCs has demonstrated therapeutic outcomes similar to those achieved with high-dose free BMSC injections for early-stage SIONFH treatment. This underscores the potency of this treatment methodology, principally as it significantly reduces the number of required cells, paving the way for large-scale clinical applications [93]. Our research team explored a specific affinity cyclic peptide, C7, for rat BMSCs through phage display technology-based biopanning. In addition, we constructed C7‑bound β‑TCP scaffolds, which exhibited increased BMSC adhesion, expansion and proliferation compared with those of pure β‑TCP scaffolds in vitro. These results suggest that C7 is effective in increasing the recruitment of BMSCs on biomaterial scaffolds, providing a novel methodology to amplify BMSC‑based bone tissue engineering therapy [94]. We subsequently demonstrated that the cyclic polypeptide D7 has a specific affinity for BMSCs and protects them against DEX-induced SIONFH in vitro [95]. Recently, silk fibroin (SF) scaffolds were coated with polydopamine (PDA) to use these active functional groups to graft short E7 peptides onto electrospun scaffolds. These composite SF-PDA-E7 electrospun scaffolds increased hydrophilicity, facilitated cell proliferation and adhesion, and increased the osteogenic differentiation of BMSCs by creating osteoinductive conditions under the synergistic effects of PDA and E7 [96]. In the case of alcohol-induced ONFH, Fu Z et al. developed a heat-sensitive nanocomposite hydrogel system featuring a secondary nanostructure that regulates gene expression and achieves sustained gene regulation in lesion cells. As the hydrogel degrades over time in vivo, the internal secondary nanostructures continue to be released. These nanoparticles carry plasmids and siRNAs into lesion stem cells, promoting the expression of B-cell lymphoma 2 (which inhibits the apoptosis of stem cells) and inhibiting the secretion of PPARγ [97].

## Concluding remarks and future perspectives

Over the past few decades, research has unequivocally established that MSCs are key players in the bone marrow microenvironment. These cells regulate bone metabolism and maintain the balance between adipogenic and osteogenic differentiation. Moreover, adipocytes differentiated in the bone marrow from mesenchymal stem cells substantially impact bone mass and angiogenesis. The increased potential for adipocytic differentiation in BMSCs, coupled with intraosseous pressure for fatty accumulation in the femoral head, is implicated in the pathogenesis of ONFH. Additionally, adipocytes secrete various adipokines, substantially affecting bone metabolism. While this review has shed light on many regulatory mechanisms governing the fates of MSCs and adipocytes in ONFH, outstanding questions remain. The specific molecular mechanism that controls the aberrant differentiation of BMSCs remains an open area of research. Likewise, studies investigating the bidirectional communication of MSCs and adipocytes within the bone marrow microenvironment are currently inadequate. Remarkably, even with previous studies indicating that bone marrow adipocytes express RANKL and promote osteoclast differentiation, the molecular mechanisms of osteoclast mediation during ONFH, especially in the context of bone remodeling, have yet to be thoroughly elucidated [28]. Unraveling the complex cellular and molecular interplay, including the interplay between MSCs and adipocytes, remains challenging owing to potential synergistic or antagonistic effects within the disrupted bone marrow microenvironment.

Generally, since MSCs and adipocytes significantly influence the bone marrow microenvironment, a deeper and more extensive investigation into the associations and interactions among BMSCs, ADMSCs, and adipocytes has potential for revealing ONFH pathogenesis and paving the way for more effective therapeutic strategies. Future research efforts should integrate multidisciplinary approaches, including molecular biology, bioinformatics, regenerative medicine, and clinical research, to develop more effective strategies for ONFH prevention and treatment. By addressing these challenges, we can move closer to developing personalized and regenerative solutions that improve patient outcomes and reduce the global burden of ONFH.

## Data Availability

Not applicable.

## Abbreviations

ONFH: Osteonecrosis of the femoral head
BMSC: Bone marrow mesenchymal stem cell
ADMSC: Adipose-derived mesenchymal stem cell
TGFβ: Transforming growth factor-beta
BMP: Bone morphogenic protein
PPARγ: Peroxisome proliferator-activated receptor-gamma
C/EBPs: CCAAT/enhancer-binding proteins
MSC: Mesenchymal stem cell
GC: Glucocorticoids
BMM: Bone marrow microenvironment
BMAT: Bone marrow adipose tissue
AT: Adipose tissue
WAT: White AT
BAT: Brown AT
RANKL: Receptor activator of NF-kB ligand
PAI-1: Plasminogen activator inhibitor-1
ALP: Alkaline phosphatase
Runx2: Runt-related transcription factor 2
COL 1: Collagen 1
OC: Osteocalcin
Adipoq: Adiponectin
PI3K: Phosphatidylinositol-3-hydroxykinase
AKT: Serine-threonine kinase
DEX: Dexamethasone
TG: Triglyceride
TC: Total cholesterol
MFAP5: Microfibril associated protein 5
SND1: Staphylococcal nuclease and tudor domain containing 1
MiRNAs: MicroRNAs
TIONFH: Trauma-induced osteonecrosis of the femoral head
hBMSCs: Human BMSCs
SIONFH: Steroid-induced ONFH
BMPR2: Bone morphogenic protein receptor 2
OPN: Osteopontin
circRNAs: Circular RNAs
ceRNA: Competing endogenous RNA
lncRNAs: Long noncoding RNAs
GILZ: Glucocorticoid-induced leucine zipper
SAA: Serum Amyloid A
MAPK: Mitogen-activated protein kinase
DMOG-treated ASCs: Dimethyloxaloylglycine-treated adipose‑derived stem cells
EVs: Extracellular vesicles
SFA: Saturated fatty acid
MUFA: Monounsaturated fatty acid
